# Excito-Secretory

**Published:** 1864-06

**Authors:** 


### "EXCITO-SECRETORY."—SINGULAR MISAPPREHENSION.

*Messrs. Editors* :—I had supposed "the real intent and meaning ,, of the phrase, *Excito-Secretory*, was, by this time, apprehended by the majority of the reading portion of the profession, but I have recently observed in an elaborate article, which need not for the present purpose be more directly indicated, a remarkable use of the term, which seems to demand a passing notice.

In the article alluded to, we are told, first, of "the excito-secretory system of nerves," and then, "the organic system," sometimes called the ganglion system, or great sympathetic nerve—the several phrases being used as synonymous.

The modern physiologist need not be told that this is a totally incorrect view, and we are led to believe the author used the terms inadvertently in the haste of composition. The author of the article alluded to, however, further confuses us by putting this "excito-secretory system" in *contrast* with the cerebro spinal system, which is wholly incorrect.

The mechanism of action, whether in the so-called cerebro-spinal, or the organic system, is identical, the resulting change depending, not on the nerve conductor, but upon the tissue reached. Histologically and physiologically, the cerebro-spinal system is as truly excito-secretory ir its action, as is the so-called organic system.

All nerves are conductors of the excito-secretory influence which is generated by molecular changes at one end of the conductor, and is manifested by molecular changes at the other end of that conductor.

There is no distinct excito-secretory system of nerves, for *all* nerves are capable of this conduction.

The terms, excito-motory, excito-secretory, excito-sensory, etc., are altogether conventional and arbitrary, tending to observe the true simplicity of the natural phenomena. J. A. A.

